# Transcriptome Sequencing Analysis of the Effect of *β*-Elemene on Colorectal Cancer from the lncRNA-miRNA-mRNA Perspective

**DOI:** 10.1155/2022/5896296

**Published:** 2022-09-13

**Authors:** Heng Deng, Shuo Chen, Xiancang Yuan, Jun Zhang

**Affiliations:** ^1^Department of Anorectal Surgery, Second Affiliated Hospital of Anhui University of Chinese Medicine, Hefei, China; ^2^The Graduate School, Guangzhou University of Chinese Medicine, Guangzhou, China; ^3^Department of Anorectal Surgery, Huainan City First People's Hospital, Huainan, China; ^4^Chinese Medicine Teaching and Research Section, Anhui University of Chinese Medicine, Hefei, China

## Abstract

*Object. β*-Elemene is an emerging antitumor Chinese medicine, but the exact mechanism of action of *β*-elemene in colorectal cancer (CRC) remains unclear. This study aimed to explore the mechanism of the lncRNA-miRNA-mRNA network in the process of *β*-elemene inhibiting CRC. *Methods*. RNA sequencing was performed on CRC cells from the control group (untreated) and the case group (*β*-elemene-treated). According to the sequencing data, we screened the differentially expressed (DE) lncRNAs, miRNAs, and mRNAs and then analyzed them by functional enrichment analyses. Through the lncRNA-miRNA-mRNA network, the key miRNAs and mRNAs involved in the process of *β*-elemene inhibiting CRC were further identified. *Results*. Totally, 607 upregulated and 599 downregulated DElncRNAs, 12 downregulated and 24 upregulated DEmiRNAs, and 3153 downregulated and 3248 upregulated DEmRNAs were identified. Through the lncRNA-miRNA-mRNA network, 3 miRNAs (miR-7109-3p, miR-4506, and miR-3182), 7 prognostic mRNAs (ALPG, DTX1, HOXD13, RIMS3, SLC16A8, SYT1, and TNNT1), and 2 key mRNAs (RIMS3 and SLC16A8) were determined to participate in the inhibitory mechanism of *β*-elemene in CRC. *Conclusion*. This study revealed for the first time that the lncRNA-miRNA-mRNA network is involved in the regulation of *β*-elemene in CRC, and these identified miRNAs and mRNAs could be new clinical prognostic biomarkers and therapeutic targets for CRC patients.

## 1. Background

Colorectal cancer (CRC) is a kind of disease with an increased morbidity year by year [1]. Many factors may contribute to the incidence of CRC, including genetics, environment, colon polyp, diet habits, and so on [2, 3]. Bloody stool is usually the earliest and commonest symptom of CRC. When it comes to the advanced stage, there will be anemia, acute peritonitis, tumor metastasis, and so on [3, 4]. In the recent 50 years, although much progress has been made in medical conditions, the 5 years survival rate of CRC still hovers around 50% due to its high recurrence [5]. At present, it is still important to explore new, safe, and effective treatment options for CRC patients.

In recent years, studies have shown that RNA with different lengths interacts with each other, especially among microRNA (miRNA), long noncoding RNA (lncRNA), and messenger RNA (mRNA), thus forming a mutual regulatory network of lncRNA-miRNA-mRNA that is important to show the interactions between RNA molecules and explain their functions [6–8]. With relatively high specificity in cells and tissues and a wide existence in various human tissues from serum and stool, miRNAs are good noninvasive biological agents for the measurement of precancerous and cancerous lesions and therapeutic targets in many diseases [9, 10]. Thus, searching for miRNAs in CRC progression and its mRNA targets could be a method to find more effective diagnostic biomarkers and treatment targets for CRC patients. Previously, some researchers analyzed the correlation between abnormally expressed miRNAs and CRC by constructing a differential miRNA bioinformatics expression map and predicted that it would affect CRC by interfering with downstream signaling pathways [11].

*β*-Elemene is a bioactive compound extracted from natural plants. As an emerging antitumor Chinese medicine, *β*-elemene has been applied in medical therapies because it could boost the effects of other antitumor Chinese medicines on lung cancer [12], breast cancer [13], colorectal cancer [14], glioma [15], osteosarcoma [16], and so on and decrease the side effects of radiotherapy and chemotherapy [17]. Previously, Chen P et al. found that by triggering ferroptosis and suppressing EMT, the *β*-elemene and cetuximab therapies were sensitive to KRAS-mutated CRC cells [14]. The other report indicates that *β*-elemene could suppress the occurrence and development of CRC in the nude mouse by affecting transplanted tumor proliferation, apoptosis, and autophagy process [18]. However, the exact mechanism between *β*-elemene and CRC has not been studied yet. Therefore, we intend to analyze the regulatory lncRNA-miRNA-mRNA mechanism of *β*-elemene-miRNA-mRNA in CRC by whole quasi-transcriptome sequencing.

This study plans to investigate the mechanism of the lncRNA-miRNA-mRNA network in the process of *β*-elemene inhibiting CRC and to further find promising therapeutic targets and prognostic biomarkers for CRC. Our findings will bring a new perspective to the CRC study and lay the therapeutic basis for the clinical treatment of CRC patients.

## 2. Materials and Methods

### 2.1. Cell Culture and Treatment

The HCT-116 cells were from the China National Collection of Authenticated Cell Culture (Shanghai, China). The cells were cultured in Dulbecco's modified Eagle's medium (DMEM) obtained from Carlsbad, CA, USA, with 2 ml glutamine and 10% Fetal bovine serum (FBS) obtained from Gibco, USA, in a humidified environment with 5% CO_2_ at 37°C. *β*-Elemene is available from Sigma. Cells treated with *β*-elemene were further used for RNA sequencing.

### 2.2. Small RNA Sequencing

We selected the control (no treatment) and case groups (*β*-elemene treatment) for RNA sequencing and pertinent analysis. Then, the RNA sequencing was performed by Origin-Biotech Inc. (Shanghai, China).

### 2.3. Identification of Differentially Expressed (DE) lncRNA, miRNA, and mRNA

Based on the RNA sequencing data, DElncRNA, DEmiRNA, and DEmRNA were identified by edgeR with a threshold of *P* < 0.05 and the absolute values of |log_2_ fold change (FC)|>1, respectively.

### 2.4. Functional Enrichment Analysis on the DElncRNA, DEmiRNA, and DEmRNA

Gene Ontology (GO) contains a set of related terms or concepts, and it describes the understanding of biology from three domains: cellular component (CC), biological process (BP), and molecular function (MF). The Kyoto Encyclopedia of Gene and Genome (KEGG) is a predictive calculation tool on the basis of relevant knowledge. Given a complete set of genes on a chromosome, it can anticipate the relevance of protein interaction networks in a variety of biological functions. Herein, the *R* package (v 3.5.1) and clusterProfiler were applied for this analysis.

### 2.5. Protein-Protein-Interaction (PPI) Network on the DElncRNA, DEmiRNA, and DEmRNA

Then, the Search Tool for the Retrieval of Interacting Genes (STRING v 11.5, https://cn.string-db.org/) tool was adopted to map PPI networks for all DElncRNA, DEmiRNA, and DEmRNA with a composite interaction score ≥0.4. Cytoscape was adopted to visualize the PPI networks. The connectivity among DElncRNA (*n* = 10), DEmiRNA (*n* = 3), and DEmRNA (*n* = 53) with high degrees was demonstrated by an independent PPI network.

### 2.6. The LASSO Analysis and Prognostic Signature Model Construction on the above 53 DEmRNAs

After the screening of the top 53 DEmRNAs from the sequencing data of *β*-elemene-treated CRC cells, we performed the least absolute shrinkage and selection operator (LASSO) regression model by the “glmnet” package of the *R* language. The relationship between the partial likelihood deviation and log (lambda, *λ*) was plotted, and the important parameters of the signature model were obtained. Moreover, we downloaded clinical samples of CRC from The Cancer and Genome Atlas (TCGA, https://tcga-data.nci.nih.gov/tcga) dataset and analyzed the prognostic values of mRNAs in clinical. First, CRC samples were classified into high-risk (*n* = 309) and low-risk (*n* = 310) groups in the risk score analysis, and the survival status of CRC samples corresponding to 15 different mRNAs was displayed. Next, the overall survival (OS) probability between two groups was compared by the Kaplan–Meier (KM, https://kmplot.com/analysis/) survival curve. Finally, the receiver operating characteristic (ROC) curve was used to compare the area under the curve (AUC) values of 1-, 3-, and 5-year survival rates of CRC, and the HR with 95% CI was calculated.

### 2.7. The Establishment of Prognostic Nomogram in CRC

To further verify the key mRNAs on the prognosis of CRC patients, the “forestplot” package was used to draw forest plots to display the results of univariate and multivariate Cox regression analyses on the above 15 mRNAs, and the corresponding *P* value and hazard ratio (HR) with 95% CI were calculated. Then, 7 mRNAs related to CRC prognosis were identified. Then, the corresponding nomogram was constructed by the “rms” package to predict the 1-, 3-, and 5-year survival rates with 7 prognostic mRNAs for CRC patients. The closer the dots are to the calibration curve, the higher the accuracy of this nomogram.

### 2.8. The Expression Verification and KM Analysis on the 7 Prognostic mRNAs

Based on the above findings, we detected their expressions in CRC tumor (*n* = 620) and normal (*n* = 10) samples in the TCGA database to identify the key mRNAs related to CRC. Then, the relations between the key mRNA expressions and OS and progression-free survival (PFS) curves were studied.

## 3. Results

### 3.1. DElncRNA, DEmiRNA, and DEmRNA from *β*-Elemene-Treated Samples

**Figure 1:**
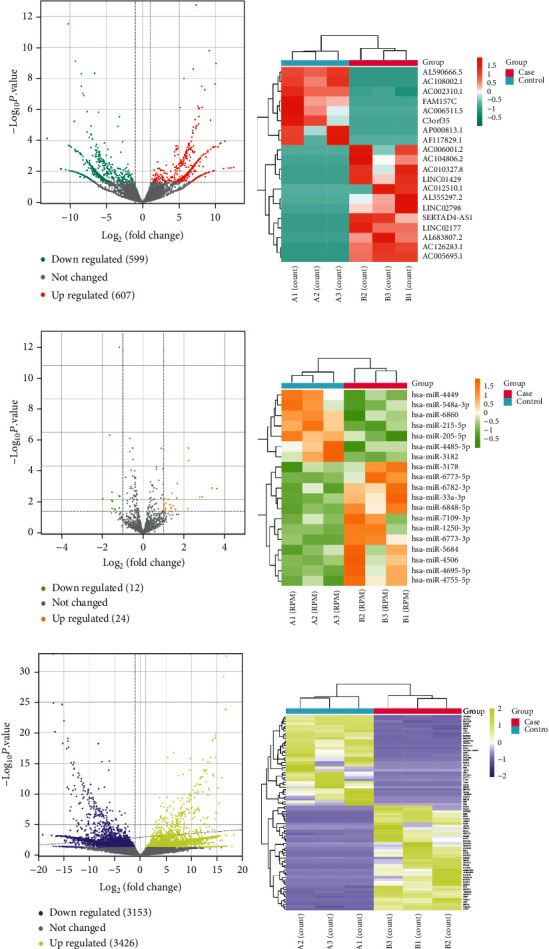
Identification of DElncRNA, DEmiRNA, and DEmRNA induced by *β*-elemene treatment in CRC. (a)-(b) Volcano map and heat map of lncRNAs. Blue stands for downregulated DEGs, red stands for upregulated DEGs, and gray stands for no significant change in genes. (c)-(d) Volcano map and heat map of miRNAs. Green stands for downregulated DEGs, orange stands for upregulated DEGs, and gray stands for no significant change in genes. (e)-(f) Volcano map and heat map of mRNAs. Yellow stands for downregulated DEGs, purple stands for upregulated DEGs, and gray stands for no significant change in genes.

First, we analyzed the DElncRNA, DEmiRNA, and DEmRNA expression profiling data from *β*-elemene-treated samples. According to the RNA-seq analysis, 607 upregulated and 599 downregulated DElncRNAs were identified and displayed by volcano and heat maps (Figures 1(a) and 1(b)), and the top 5 upregulated and downregulated DElncRNAs are demonstrated in Table 1. As shown in Figures 1(c) and 1(d), we identified 12 downregulated and 24 upregulated DEmiRNAs, and the top 5 of each group are shown in Table 2. Besides, 3153 downregulated and 3248 upregulated DEmRNAs are displayed in Figures 1(e) and 1(f), and the top 5 DEmRNAs of them are also shown in Table 3.

### 3.2. The Results of GO Term and KEGG Pathway Enrichment Analyses

**Figure 2:**
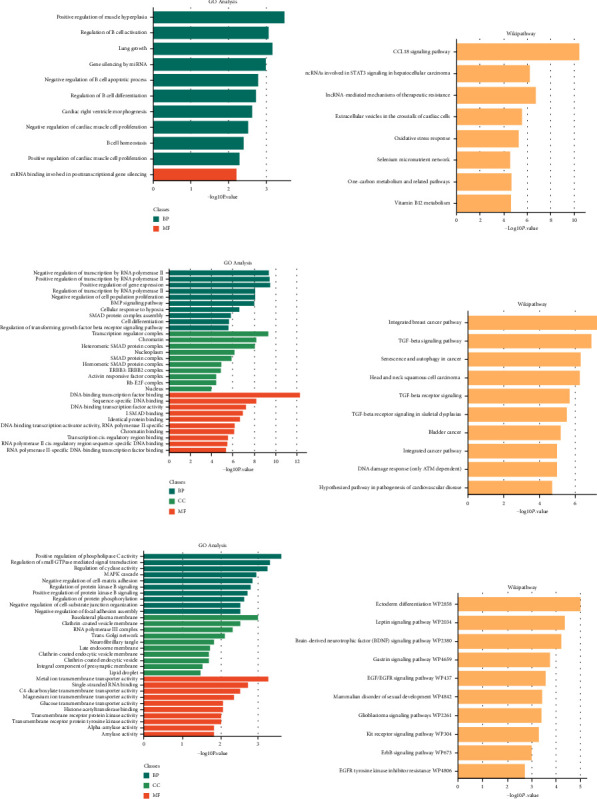
The GO and KEGG enrichment analyses on DElncRNAs, DEmiRNAs, and DEmRNAs. (a) The GO analysis of lncRNAs. (b) The KEGG pathway enrichment analysis of lncRNAs. (c) The GO analysis of miRNAs. (d) The KEGG pathway enrichment analysis of miRNAs. (e) The GO analysis of mRNAs. (f) The KEGG pathway enrichment analysis of mRNAs.

In GO analysis, DElncRNAs were enriched in positive regulation of muscle hyperplasia, regulation of B cell activation, lung growth (BP), and mRNA binding involved in posttranscriptional gene silencing (MF, Figure 2(a)). DEmiRNAs were enriched in negative regulation of transcription by RNA polymerase II, positive regulation of gene expression (BP), transcription regulator complex, chromatin, heteromeric SMAD protein complex (CC), DNA-binding transcription factor binding, and sequence-specific DNA binding (MF, Figure 2(c)). DEmRNAs were enriched in positive regulation of phospholipase C activity (BP), basolateral plasma membrane (CC), clathrin-coated vesicle membrane, and RNA polymerase III complex (MF, Figure 2(e)). In the KEGG pathway analysis, DElncRNAs were related to the CCL18 signaling pathway, ncRNAs involved in STAT3 signaling in hepatocellular carcinoma, lncRNA mediated mechanisms of therapeutic resistance (Figure 2(b)), DEmiRNAs were related to the integrated breast cancer pathway, the TGF-beta signaling pathway, senescence, and autophagy in cancer (Figure 2(d)), and DEmRNAs were related to the ectoderm differentiation, the leptin signaling pathway, and so on (Figure 2(f)).

### 3.3. The lncRNA-miRNA-mRNA Coexpression Network Analysis

**Figure 3:**
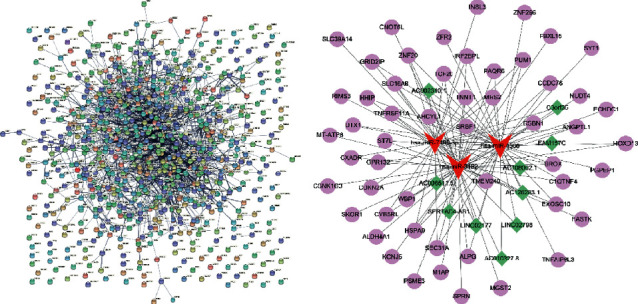
The lncRNA-miRNA-mRNA coexpression network analysis. (a) The PPI network of the DERmNAs. (b) The lncRNA-miRNA-mRNA network of RNAs with top degrees in each category.

Next, we constructed a PPI network of the DERNAs with the Cytoscape tool (Figure 3(a)). According to the degrees in each group, DERNAs with high degrees (composite interaction score ≥0.4) in each group were selected for the construction of the lncRNA-miRNA-mRNA network, including 10 DElncRNAs, 3 DEmiRNAs, and 53 DEDmRNAs. As demonstrated in Figure 3(b), miR-7109-3p, miR-4506, and miR-3182 were closely related to ambient DElncRNAs and DEmRNAs, which indicated that miR-7109-3p, miR-4506, and miR-3182 had the potential to be clinical biomarkers or therapeutic targets in CRC.

Nodes represent genes, and edges represent interactions between genes. The green nodes represent the lncRNA, the red nodes represent the miRNA, and the purple nodes represent the mRNA.

### 3.4. The LASSO Analysis and Prognostic Signature Model Construction of the 53 DEmRNAs

**Figure 4:**
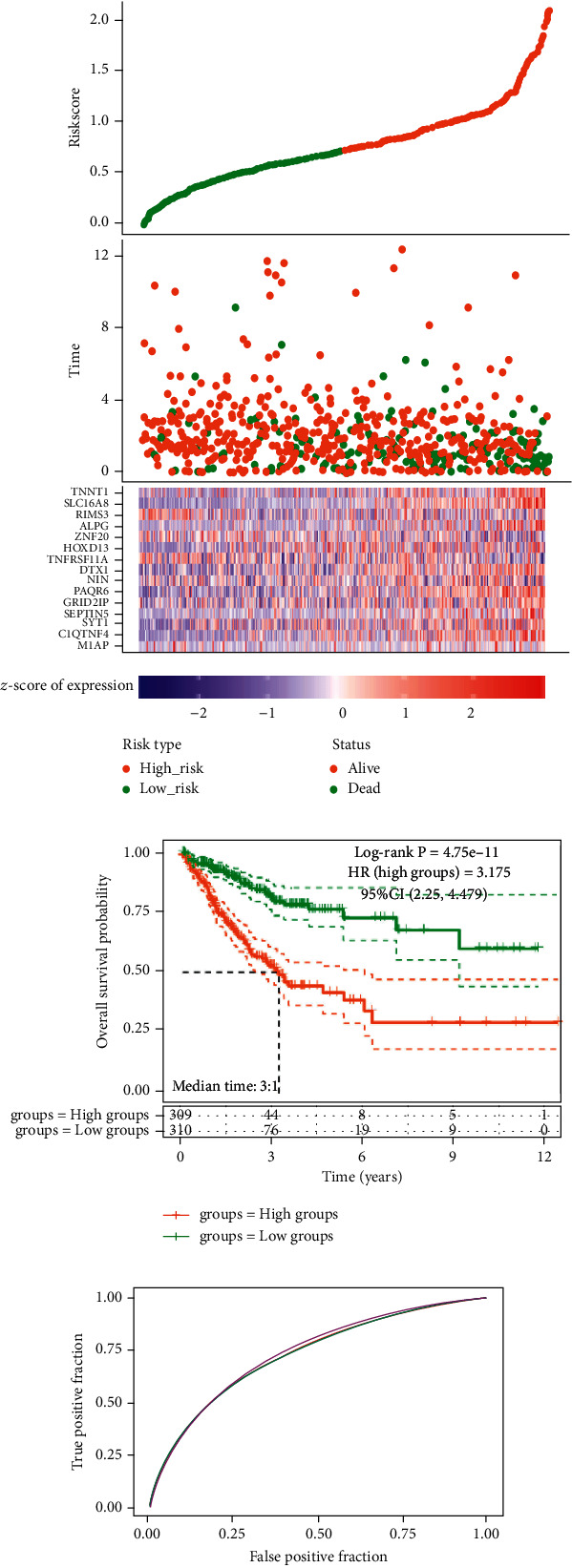
The LASSO analysis and prognostic signature model construction on the 53 mRNAs. (a)-(b) The LASSO regression mode analysis on the 53 mRNAs. (c) The risk score evaluation of CRC patients (top). The corresponding survival time of each patient (middle). The Z-score of expression of 15 candidate prognostic mRNAs (bottom). (d) Prognostic analysis of high-risk and low-risk groups. (e) The ROC curve analysis.

The LASSO regression analysis was first performed on the above 53 DEmRNAs to determine the optimal parameter number of the signature model (Figures 4(a) and 4(b), each line represented a DEmRNA). Then, CRC samples in the TCGA database were divided into high- (*n* = 309) and low-risk groups (*n* = 310), and it was found that the 15 mRNAs, M1AP, C1QTNF4, SYT1, SEPTIN5, GRID2IP, PAQR6, NIN, DTX1, TNFRSF11 A, HOXD13, ZNF20, ALPG, RIMS3, SLC16A8, and TNNT1, had the potential to be prognostic biomarkers in CRC (Figure 4(c)). In the KM survival curve, the median time of the two groups was 3.1 years, and the OS probability of the high-risk group was poor, and its HR was 3.175 (>1), indicating that the model was a risk model (Figure 4(d)). In addition, the AUC values in the ROC curve were all greater than 0.7, suggesting a good prognostic prediction ability of the model (Figure 4(e)).

### 3.5. The Identification of mRNAs with Prognostic Value in CRC

**Figure 5:**
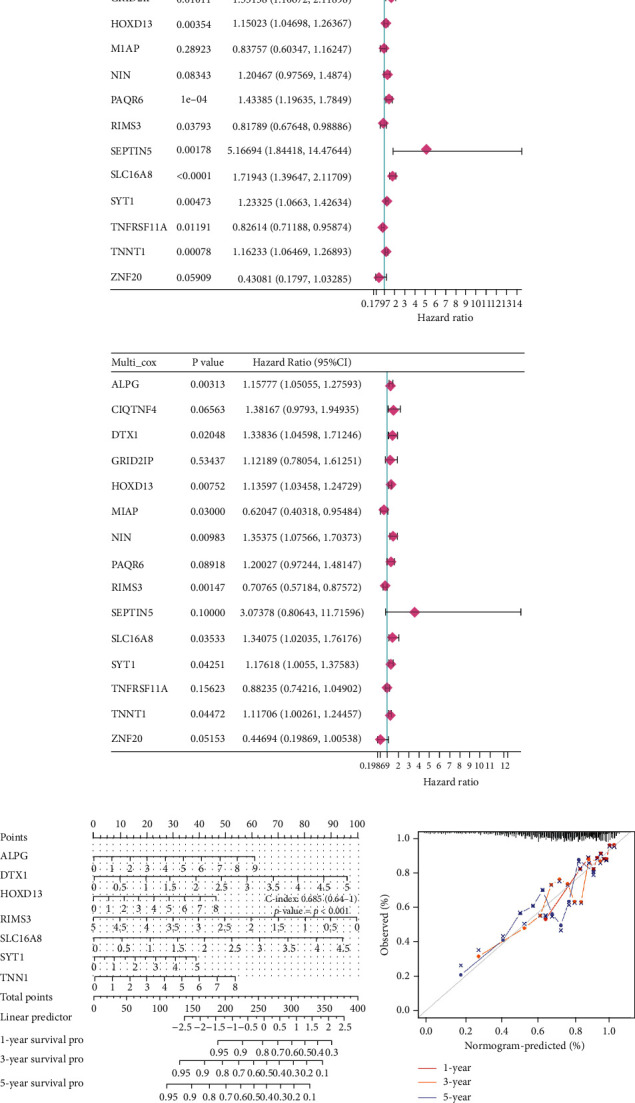
The prognostic nomogram with prognostic mRNAs. (a)-(b) Univariate and multivariate Cox regression analyses. (c) The prognostic nomogram with prognostic mRNAs. (d) The dotted line is the ideal calibration curve of the nomogram.

To investigate the mRNAs with prognostic values in CRC, we performed univariate and multivariate Cox regressions on the above 15 DEmRNAs. Under the premise of *P* < 0.05, it was found that ALPG, DTX1, HOXD13, RIMS3, SLC16A8, SYT1, and TNNT1 had a close relationship with the prognosis of CRC patients (Figures 5(a) and 5(b)). Subsequently, a nomogram on these genes was designed to predict the 1-, 3-, and 5-year survival rates of CRC patients (Figure 5(c)). As shown in Figure 5(d), the different dots were relatively close to the calibration curve, indicating that the prognostic model had good predictive ability.

### 3.6. RIMS3 and SLC16A8 Were the Key mRNAs Related to CRC in *β*-Elemene Treatment

**Figure 6:**
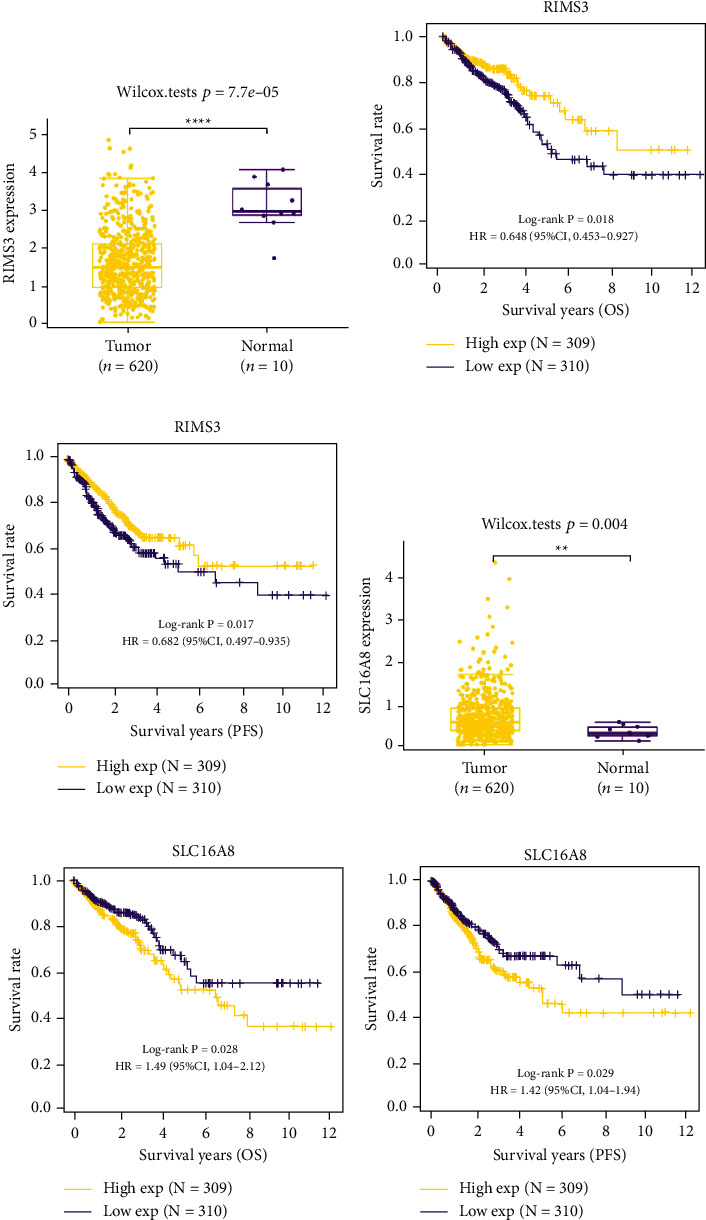
RIMS3 and SLC16A8 were the key mRNAs related to CRC in *β*-elemene treatment. (a) RIMS3 significantly downregulated in CRC samples. (b)-(c) The Kaplan–Meier survival curve showing that lower expression levels of RIMS3 were significantly associated with shorter OS and PFS in CRC. (d) SLC16A8 upregulated in tumor samples. (e)-(f) The Kaplan–Meier survival curve showing that higher expression levels of RIMS3 were significantly associated with shorter OS and PFS in CRC. ^*∗∗*^*P* < 0.01, ^*∗∗∗∗*^*P* < 0.0001.

To further explore the exact mRNAs related to CRC in *β*-elemene treatment, we detected the expressions of the 7 prognostic mRNAs in CRC tumor and normal samples. After detection, it was found that RIMS3 was significantly increased in normal samples, while SLC16A8 was substantially upregulated in tumor samples (Figures 6(a) and 6(d)), which indicated that RIMS3 was a suppressor gene while SLC16A8 was an oncogene in CRC. In the OS and PFS analyses on them, low expression of RIMS3 represented a poor survival rate, while low expression of SLC16A8 represented a relatively good survival rate (Figures 6(b), 6(c), 6(e), 6(f)).

## 4. Discussion

In recent years, the lncRNA-miRNA-mRNA network has been applied in the mechanism study of various diseases. For example, Li et al. found a novel lncRNA-miRNA-mRNA signature that predicted recurrence and disease-free survival in cervical cancer [19]. Cao et al. found through miR-206, lncRNA-RMRP increased bladder cancer development [20]. Besides, some researchers demonstrated lncRNA-CDC6 might act as a ceRNA to facilitate breast cancer development by sponging miRNA-215 [21]. In this study, we also tried to investigate the exact mechanism in *β*-elemene inhibiting CRC progression from the aspect of the lncRNA-miRNA-mRNA network.

Through deep sequencing, high-throughput screening, and microarray technology, many dysregulated miRNAs were found in cancer cells. Due to the tissue specificity of miRNA regulation, miRNAs have much potential to be biomarkers in cancer diagnosis, treatment, and prognosis [22]. In this study, we identified 1026 DElncRNAs, 6401 DEmRNAs, and 36 DEmiRNAs in *β*-elemene-treated HCT116 cells using RNA sequencing. Then, these DERNAs were analyzed by functional analyses and PPI networks. Enrichment analysis showed that DElncRNAs were associated with the CCL18 signaling pathway. Ruixue Yuan et al. showed that high CCL18 levels can be an independent biomarker for predicting better survival in CRC patients [23]. The DEmiRNAs were related to the TGF-beta signaling pathway. TGF-beta signaling is one of the important cellular pathways that plays a key role in tissue maintenance. Nan et al. mentioned that LINC00941 plays an important role in metastatic CRC by activating the TGF-*β*/SMAD2/3 axis [24]. DEmRNAs are related to the leptin signaling pathway. Leptin is an activator of cell proliferation, an antiapoptotic molecule, and an inducer of cancer stem cells in many cell types. Studies have shown that the stringent binding affinity of leptin/Ob-R and the overexpression of leptin/Ob-R and its targets in cancer cells make it a unique drug target for the prevention and treatment of CRC, especially in obese colorectal patients [25]. In the PPI network, we selected the top RNAs in each group and observed their mutual relations, including miR-7109-3p, miR-4506, and miR-3182. At present, there are few reports on miR-7109-3p. One study highlights LINC00973-miR-7109-Siglec-15 function in the immune evasion of clear-cell renal cell carcinoma [26]. Nagy discovered miR-4506 was differentially expressed in adenoma compared to normal both in CRC tissue and plasma samples [27]. Moreover, it was found that the biological functions and potential mechanisms of miR-3182 were altered after Ganoderic acid treatment in CRC [28]. The 3 miRNAs all have the potential to be clinical biomarkers in CRC.

Besides, we focused on the mRNA associated with *β*-elemene and CRC. Through LASSO, risk score evaluation, and Cox analyses, 7 prognostic mRNAs, ALPG, DTX1, HOXD13, RIMS3, SLC16A8, SYT1, and TNNT1, were identified and applied to design the predictive nomogram for CRC prognosis, and the nomogram was proved to have a good prediction ability. In the previous research, few studies were conducted on these genes. In a study on TNNT1, researchers demonstrated that TNNT1, a target of miR-873, is related to CRC prognosis [29]. Herein, our findings show that the 7 mRNAs could be the prognostic biomarkers for CRC patients.

Furthermore, two key mRNAs related to CRC were identified by expression and KM analysis, namely, RIMS3 and SLC16A8. According to the public database, RIMS3 was a suppressor gene in CRC, while SLC16A8 was an oncogene in CRC. Regulating synaptic membrane exocytosis 3 (RIMS3) is supposed to enable transmembrane transporter binding activity and participate in the calcium ion-regulated exocytosis of neurotransmitters. Currently, it is suggested that RIMS3 is a gene related to autism. Functional investigations on RIMS3 variations like p.E177 A deepen our understanding of synaptic proteins' function in autism pathogenesis [30]. Additionally, solute carrier family 16 member 8 (SLC16A8), alias MCT3 and REMP, belongs to a family of proton-coupled monocarboxylate transporters regulating lactate transport across cell membranes [31]. The study of Klipfel L demonstrates the loss of transport function of the hypomorphic allele in the SLC16A8 gene and offers a methodological framework for the investigation of other SLC16A8 alleles linked to age-related macular degeneration [32]. Currently, there are no reports about these two genes in CRC development. Based on our study, we conclude that RIMS3 and SLC16A8 are the key mRNAs involved in the *β*-elemene treatment in CRC progression, which certainly reveals the inhibitory mechanism of *β*-elemene in CRC. The study still has limitations. First, the expression profiles given by differential expression need to be validated with clinical samples. Second, further experimental studies are required to confirm the function of the lncRNA-miRNA-mRNA network in the inhibition of CRC progression by *β*-elemene.

To sum up, this study reveals the lncRNA-miRNA-mRNA network in the inhibitory function of *β*-elemene in CRC. These identified differentially expressed mRNAs (RIMS3 and SLC16A8) and miRNAs (miR-7109-3p, miR-4506, and miR-3182) all could be promising clinical biomarkers or targets in CRC diagnosis, treatment, and prognosis.

## Figures and Tables

**Table 1:** Top 5 increased and decreased DElncRNAs.

Gene ID	Transcript	Gene name	Status	*P* value
ENSG00000225791	ENST00000667019	TRAM2	Increased	1.12E-09
ENSG00000285517	ENST00000649390	Novel gene	Increased	2.35E-08
ENSG00000271853	ENST00000497086	Novel gene	Increased	5.04E-06
ENSG00000258525	ENST00000661204	Novel gene	Increased	1.64E-10
ENSG00000179818	ENST00000654878	PCBP1	Increased	5.46E-08
ENSG00000260528	ENST00000563357	FAM157 C	Decreased	3.20E-08
ENSG00000247679	ENST00000656340	Novel gene	Decreased	5.10E-09
ENSG00000257261	ENST00000663959	Novel gene	Decreased	7.71E-10
ENSG00000232931	ENST00000660641	LINC00342	Decreased	5.75E-06
ENSG00000267432	ENST00000663269	DNAH17	Decreased	2.96E-12

**Table 2:** Top 5 increased and decreased DEmiRNAs.

miRNA	Status	logFC	*P* value
hsa-miR-6773-3p	Increased	3.61676331	0.002245373
hsa-miR-1250-3p	Increased	3.379810682	0.002053361
hsa-miR-6848-5p	Increased	2.780991902	0.00707793
hsa-miR-4506	Increased	2.226485819	8.45E-06
hsa-miR-7109-3p	Increased	2.184563759	0.034727373
hsa-miR-548a-3p	Decreased	−1.378214532	0.040108511
hsa-miR-4449	Decreased	−1.398551955	0.037313819
hsa-miR-4485-5p	Decreased	−1.50328227	0.01272798
hsa-miR-3182	Decreased	−1.644656839	1.41E-06
hsa-miR-215-5p	Decreased	−1.987458083	0.008964197

**Table 3:** Top 5 increased and decreased DEmRNAs.

Gene ID	Transcript	Gene name	Status	*P* value
ENSG00000129657	ENST00000430767	SEC14L1	Increased	6.56E-20
ENSG00000144580	ENST00000542068	CNOT9	Increased	1.13E-11
ENSG00000161016	ENST00000533397	RPL8	Increased	3.45E-09
ENSG00000149782	ENST00000325234	PLCB3	Increased	7.20E-30
ENSG00000145425	ENST00000512690	RPS3A	Increased	1.55E-24
ENSG00000084774	ENST00000403525	CAD	Decreased	1.34E-33
ENSG00000167881	ENST00000539137	SRP68	Decreased	7.75E-12
ENSG00000124532	ENST00000274747	MRS2	Decreased	2.38E-19
ENSG00000197713	ENST00000354506	RPE	Decreased	1.61E-11
ENSG00000254093	ENST00000519088	PINX1	Decreased	2.39E-18

## Data Availability

The datasets used to support the findings of this study are available from the corresponding author upon request.
